# The Interplay between Antioxidants and the Immune System: A Promising Field, Still Looking for Answers

**DOI:** 10.3390/nu12061550

**Published:** 2020-05-26

**Authors:** Giulio Francesco Romiti, Bernadette Corica, Valeria Raparelli, Stefania Basili, Roberto Cangemi

**Affiliations:** 1 Department of Translational and Precision Medicine—Sapienza University of Rome, 00161 Rome, Italy; giuliofrancesco.romiti@uniroma1.it (G.F.R.); bernadette.corica@uniroma1.it (B.C.); stefania.basili@uniroma1.it (S.B.); 2 Department of Experimental Medicine, Sapienza University of Rome, 00161 Rome, Italy; valeria.raparelli@uniroma1.it

Modulation of the immune response has long been proposed as a therapeutic target in several widespread diseases, including cancer, autoimmune disorders, cardiovascular diseases, and also during the dysregulated response phase towards a systemic infection [1,2]. Potential benefits of immunomodulation depend on the interplay between immune cells and the disease progression, as observed in atherosclerosis [3,4], heart failure [5] and different types of neoplasms, although with different levels of evidence. Immunosuppression is also an established cornerstone for the treatment of autoimmune disorders, including systemic lupus erythematosus, rheumatoid arthritis, and other connective tissue diseases. However, most of the drugs targeting the immune system carry several side effects, one of the most important being the increased risk of infection, a logical consequence of the reduced activity of the immune system. 

In the unstoppable search for the “silver bullet”, which aims to maximize the efficacy and minimize side effects, Malaguarnera provides a thorough review of the effects of Resveratrol [6], focusing on the mechanisms behind the complex interplay between these molecules and the immune cells. The antioxidant effects of Resveratrol have been known for decades [7], and its use has been investigated in different clinical contexts. Resveratrol is found in red wine and has been speculated to be the primary factor responsible for the so-called "French Paradox", although with no conclusive evidence. [8] As elegantly summarized by Malaguarnera, Resveratrol may express its action through a complex interplay with Sirtuins [6]; however, the clinical application has been limited by its low oral bioavailability, which reduces its effectiveness [9]. A potential role as an immunomodulating agent has been theorized in recent decades, although, to date, extensive and definitive data on its clinical efficacy have been lacking. 

Research on the complex interplay between oxidative stress, the immune system, and agents targeting both pathways is gathering growing interest among the scientific community. Several other agents have been studied, such as steroids, Vitamin C, and Vitamin D, especially in the field of cardiovascular and infectious diseases [10,11,12,13]. The intensive care setting has been one of the most investigated scenarios in the search for immunomodulating agents, with Vitamin C failing to show a significant effect on improving outcomes in sepsis [12,13]. Steroids are the most common immunosuppressive agents used to improve symptoms in auto-immune disorders, in which the immune response is increased by the production of inflammatory cytokines and auto-antibodies. The immunomodulatory effect of steroids has been postulated, and their use in the setting of intensive care has been widely studied, with conflicting results [14,15]. Currently, steroids find application in refractory septic shock therapy [16].

Resveratrol may represent, in this context, an alternative approach. Several experiments in animal models have shown potential efficacy in the prevention and treatment of different diseases [17,18]. Interest in this molecule has increased due to its natural presence in many different foods, including peanuts, red grapes, and red wine [19,20]. However, its relatively low oral bioavailability, along with pharmacokinetics issues and the quality of the commercially-available supplements, have somewhat limited the application in clinical practice [9,21]. Translation of the results obtained in animal models has also been slowed by the heterogeneity in dosage protocols among human studies, and the optimal dose for clinical application is far from being clarified [22]. 

These issues are shared by many other nutraceuticals and antioxidants [23,24,25]. To date, the lack of standardized formulation and dose regimens, as well as low numbers of high-quality studies in humans, limit the evidence available on the clinical use of these substances, the commercialization of which is often not under the control of international regulatory agencies.

Between the promising findings of pre-clinical studies and the problems arising in clinical practice, only well-designed and rigorous clinical studies can provide definitive answers on the efficacy and safety of these compounds. While in vitro evidence suggests potential room in many clinical settings, including immune function [26], it is conceivable that the identification of more specific clinical scenarios will help in determining the true extent of the expected benefit of these drugs, including Resveratrol. The immune response represents a fascinating but complex target; to date, ongoing trials on Resveratrol are mainly focused on cardiovascular and metabolic diseases [27,28,29]. Neoplasia represents another setting in which Resveratrol was tested, with different results according to cancer types [30]. Looking at the interplay with the immune system may provide a new perspective to evaluate different clinical responses to antioxidants, but only thorough and well-conducted mechanistic studies will elucidate whether the supposed effects of these compounds—including Resveratrol—may translate into clinical results. 
